# Rare Combination of Chyloperitoneum Secondary to Primary Small-Bowel Volvulus

**DOI:** 10.7759/cureus.53379

**Published:** 2024-02-01

**Authors:** Diogo Galvão, Rui Bettencourt, Ana Cláudia Soares, Inês Bagnari, Joana Bonança

**Affiliations:** 1 Surgery, Hospital de Santo Espírito da Ilha Terceira, Angra do Heroísmo, PRT

**Keywords:** mechanical intestinal obstruction, primary volvulus, small bowel, chyloperitoneum, chylous ascites

## Abstract

Chylous ascites is the exudation from lymphatic content to the peritoneum and is a rare situation that mostly occurs following medical causes like neoplasms or cirrhosis. However, trauma to the lymphatic system due to compression by masses or altered anatomy can be a trigger too. We describe a rare combination of a primary small bowel volvulus in a young healthy adult causing chylous ascites.

Obstruction caused by a primary small bowell volvulus can re-arrange the lymphatics anatomy increasing their flow pressure which can lead to rupture and leak. This is an emergent scenario that needs to be addressed quickly because of bowel ischaemia. CT scan is the gold standard to expedite diagnosis and go to surgical treatment.

Although it can be an impactful finding, treatment of the cause behind chylous ascites results in complete resolution without any bowel resection.

## Introduction

Chylous ascites is a rare finding [1] that results from lymph leakage into the peritoneal cavity secondary to trauma or rupture of lymphatic vessels [2]. Two-thirds of the cases are secondary to congenital lymphatic malformations, neoplasms, and cirrhosis and the remaining are due to local trauma or iatrogenic lesion [3]. A primary small bowel volvulus is an aberrant rotation of a small bowel segment along the axis of its mesentery [4] and it’s a rare cause of chylous ascites.

We describe a rare cause of intestinal obstruction presenting with chylous ascites in an untouched abdomen of a healthy young patient.

## Case presentation

A 22-year-old male patient was admitted to the Emergency Department (ED) with sudden onset of severe and acute abdominal pain with four hours of progression. He felt a colicky, diffuse, and non-irradiating abdominal pain. He reported two bloodless vomitings, regular bowel movement the day before and no urinary complaints. He was otherwise healthy, without a surgical background.

The patient was restless on physical examination. On palpation, the abdomen was soft but generally painful. Blood pressure was 151/106 mmHg, pulse 74 beats/minute and temperature 36.0ºC. Blood samples revealed white blood cell count of 19.3x10^9/L with normal range values for the rest of the parameters.

**Figure 1:**
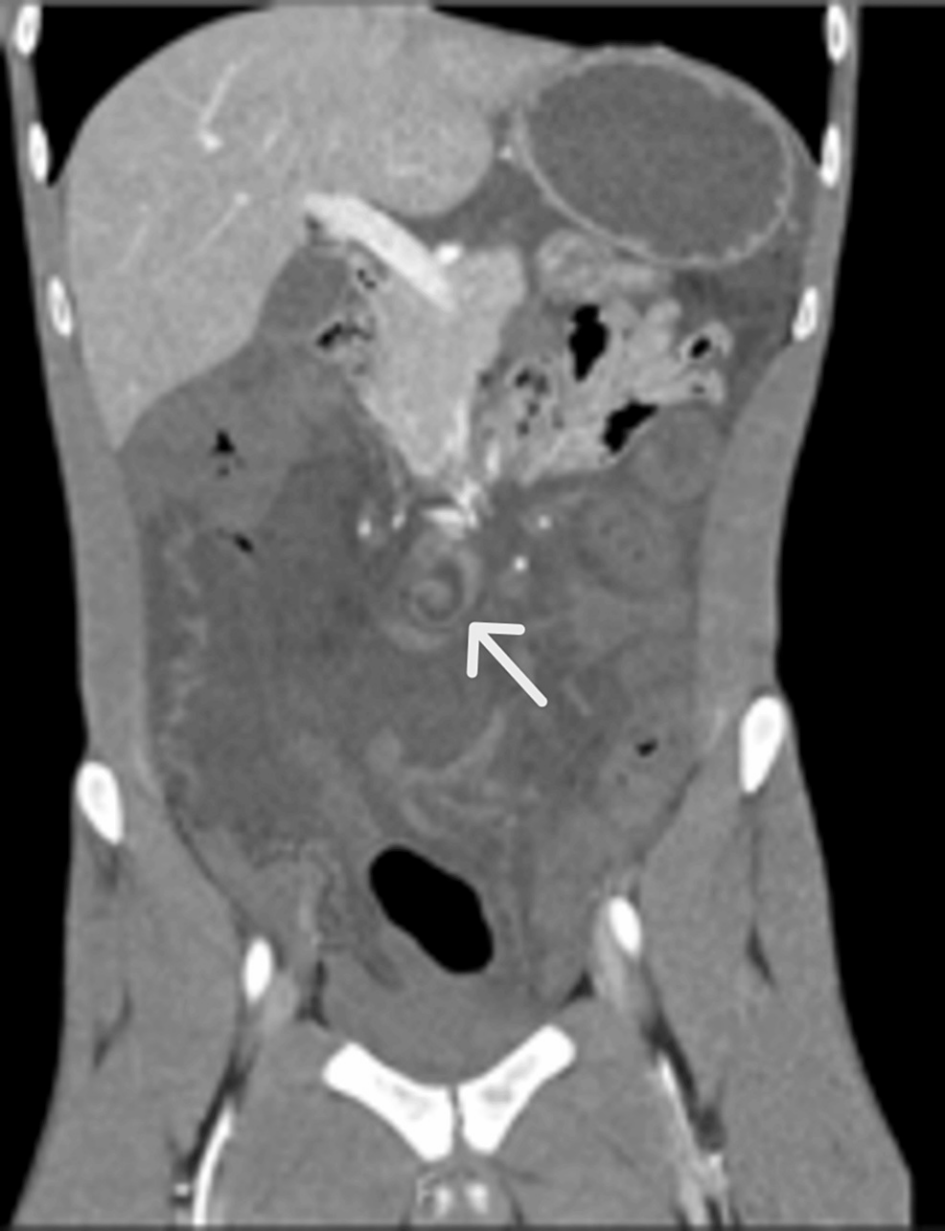
CT Scan coronal view: Whirlpool sign (arrow)

**Figure 2:**
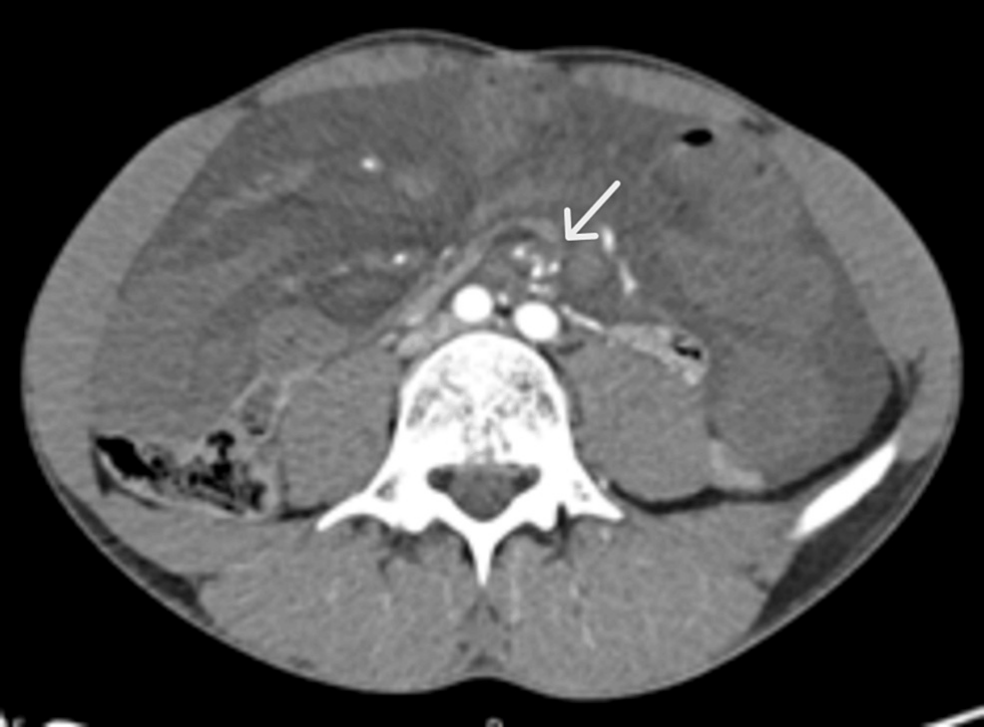
CT Scan axial view: Whirlpool sign (arrow)

A computed tomography (CT) scan of the abdomen and pelvis revealed moderate volume ascites with greater expression in the lower quadrants and the whirl sign of the mesenteric vascular structures (Figures 1, 2), with no permeability of the mesenteric vein or artery downstream. Marked asymmetry of the parietal enhancement of the intestinal loops, without parietal enhancement of the small bowel loops distal to the referred rotation, was observed.

**Figure 3:**
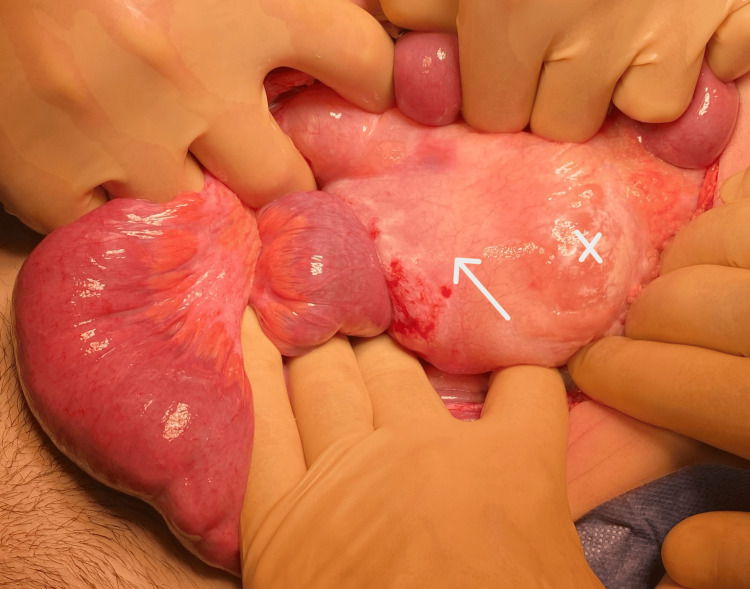
Intra-operative image of chylous mesenteric infiltration (arrow) and mesenteric milky adenopathy (x)

The patient underwent exploratory laparotomy. A clear, milky fluid was present in all quadrants of the abdominal cavity. Abdominal exploration revealed a small bowel volvulus with a 180º rotation around the mesenteric axis. The intestinal loops had a violaceous ischemic appearance. There was also a mesenteric infiltration of several areas of the small intestine by the same substance and mesenteric adenopathies with the same milky appearance (Figure 3). The intestine was distorted and, after a few minutes of warming with heated normal saline, it gained a normal appearance and peristalsis, indicating tissue viability. An excisional biopsy of one mesenteric adenopathy was performed, and collection of ascitic fluid for biochemical and cytology assays. No other relevant findings were found intraoperatively. An aspiration drain was left, and the abdominal wall was closed by layers.

The ascitic fluid was sterile and revealed triglycerides of 641 mg/dl supporting the diagnosis of chylous ascites [2]. Cytology was negative for the presence of neoplastic cells and histology of adenopathy revealed the presence of reactive lymph node. 

The patient's postoperative period was uneventful. The drainage was clear from the first postoperative day and the patient went home on the fourth postoperative day.

## Discussion

Our case is an unusual combination, because the chylous ascites was caused by a rare primary small bowel volvulus in an adult. The literature for mesenteric lymphatic infiltration and small bowel volvulus is scarce and focuses primarily on paediatric cases or patients with a surgery or intra-abdominal radiotherapy past [5-9]. Only a few case reports and small case series [10-12] describe this unique combination. 

The clinical presentation of an intestinal volvulus is challenging because it’s not specific or different from other acute abdomen causes. In our case, sudden abdominal pain unresponsive to analgesia suggested an acute abdomen despite the unremarkable blood analysis. Although the abdominal palpation didn’t reflect any tenderness, the overall clinical impression supported the use of the CT scan with intravenous contrast. The whirl sign (Figures 1, 2) and the absence of intravenous contrast in the loops downstream of this area increased the suspicion of volvulus with bowel ischaemia, and forwarded the decision to do an urgent exploratory laparotomy [13]. According to the World Society of Emergency Surgery (WSES) 2017 guidelines on adhesive small bowel obstruction, CT scan has approximately 90% accuracy in predicting strangulation and the need for urgent surgery [14].

The patient was otherwise healthy, and we didn’t find any adhesion bands or other findings on the exploratory laparotomy that could justify a secondary small bowel volvulus. Thus, a primary small bowel volvulus was diagnosed as the cause of the chylous ascites. The recovery of intestinal viability after distortion described in our case report, even in the presence of ascites and chylous mesenteric infiltration, is in line with the literature review by Harino et al*. *[6]. They gathered all the cases of chylous ascites associated with strangled ileus and found 22 in the Japanese and English literature. Only eight patients had no history of previous surgery, no bowel resection was needed for anyone, and recovery of intestinal viability occurred in 100% of patients after distortion or release of the internal hernia [6]. 

Athanasiadis et al.[9] proposed that the presence of chylous ascites with intestinal strangulation due to internal hernia is an innocuous finding. Hayama et al. [12] theorize that chylous ascites in a patient with volvulus or internal hernia is innocuous and it may reflect the patency of the mesenteric vessels. In our case and those by Harino et al. [6] there was no intestinal necrosis either corroborating those hypotheses.

Thus, one can say that in the presence of an intestinal volvulus, the finding of chylous ascites should not be understood as a poor prognostic feature despite its unusual appearance. 

## Conclusions

A primary small bowel volvulus is a rare cause of chylous ascites. An expedited diagnosis is crucial for a timely treatment because of the risk of bowel ischaemia. Contrast-enhanced CT scan is the gold standard to diagnose this condition.

The authors acknowledge that the presence of chylous ascites can be impactful and mimic a more severe situation, due to the rarity and look of this finding. However, we emphasize that it shouldn’t change the approach of the condition behind it, because there is a complete resolution in 100% of patients without bowel resection when the cause is treated.
